# Rubella Virus Genotype 1E in Travelers Returning to Japan from Indonesia, 2017

**DOI:** 10.3201/eid2409.180621

**Published:** 2018-09

**Authors:** Daiki Kanbayashi, Takako Kurata, Yuka Nishino, Fumi Orii, Yuki Takii, Masaru Kinoshita, Toshitake Ohara, Kazushi Motomura, Takahiro Yumisashi

**Affiliations:** Osaka Institute of Public Health, Osaka, Japan (D. Kanbayashi, T. Kurata, K. Motomura, T. Yumisashi);; Osaka Prefectural Government Department of Health and Medical Care, Osaka (Y. Nishino, F. Orii, Y. Takii, M. Kinoshita);; Osaka Prefectural Government Ikeda Healthcare Center, Osaka (T. Ohara)

**Keywords:** rubella, rubella virus, genotype 1E, lineage 2, Indonesia, viruses, Japan

## Abstract

Although rubella is epidemic in Indonesia, the phylogenetic profile of circulating rubella virus strains has not been clarified. In 2017, rubella virus was detected in 2 travelers who returned from Indonesia to Japan. These strains were classified into genotype 1E lineage 2, which may be an indigenous strain in Indonesia.

Rubella is a mild contagious disease caused by the rubella virus, genus *Rubivirus*, family *Togaviridae* (*1*). Fetal death or congenital rubella syndrome (CRS) can occur when infection arises in pregnant women (*1*). Rubella infections and CRS cases have declined in many countries because of vaccination (*2*); however, an estimated 110,000 CRS cases occurred globally in 2010, with almost half developing in Southeast Asia because routine immunization programs against rubella virus had scarcely been introduced in these countries at that time (*3*,*4*). As of 2016, of the 11 countries in Southeast Asia, 8 (Bangladesh, Bhutan, Maldives, Myanmar, Nepal, Sri Lanka, Thailand, and Timor-Leste) had introduced routine immunization (*5*). However, large epidemics still exist in Southeast Asia, mainly in India and Indonesia, which had not introduced routine immunization as of 2016 (*5*). In addition, CRS cases in Indonesia were the highest worldwide in 2016 (*5*). Contrarily, only 1 sequence of the virus in Indonesia was registered in GenBank, from a patient who returned to the United States in 2011 (Hendersonville.NC.USA/15.11, accession no. JX477651). Although these rubella-endemic countries greatly affect the efforts of neighboring countries to control the virus, genetic information of epidemic strains in Southeast Asia remains unclear.

In October 2017, a 29-year-old man in Japan experienced a slight fever and sore throat. He had traveled to Jakarta, Indonesia, in late September, 14 days before symptom onset. He was not previously vaccinated against rubella virus. On day 4 after onset, rashes appeared on his body. Testing by real-time reverse transcription PCR did not detect the measles virus genome, but it detected rubella virus genome via throat swab sample collected on day 7 after onset (*6*). His illness was diagnosed as rubella; we strongly suspected that he acquired the infection in Indonesia, because the incubation period of rubella virus is ≈14 days and Japan has had no domestic rubella epidemic since 2013.

We amplified the E1 protein-coding region genome of the virus and sequenced the molecular window region (739 nt) (*7*). We classified this rubella strain into genotype 1E and deposited it into GenBank (RVs/Osaka.JPN/41.17[1E], accession no. LC333396). We generated a phylogenetic tree including 61 strains using the maximum-likelihood method (Figure); it revealed that rubella virus can be classified into 5 distinct lineages (L0–L4), as previously described (*7*,*8*). 1E-L1 strains are mainly detected in China and Russia. 1E-L2 strains are mainly detected in or imported from Malaysia, China, and Japan. 1E-L3 strains are detected in or imported from African countries, such as the Democratic Republic of the Congo and Tunisia. 1E-L4 strains are detected in Sudan, Yemen, and Uganda. Both RVs/Osaka.JPN/41.17[1E] and Hendersonville.NC.USA/15.11 belonged to 1E-L2; these sequences were closely related to the recently identified 1E-L2 sequences. We also detected RVs/Yokohama.JPN/3.17[1E] in a traveler who returned to Japan from Indonesia in January 2017 (deposited in GenBank under accession no. LC215401) who had contact with a local rubella patient. Our findings indicate that 1E-L2 strains may circulate as indigenous strains in Indonesia. 

**Figure:**
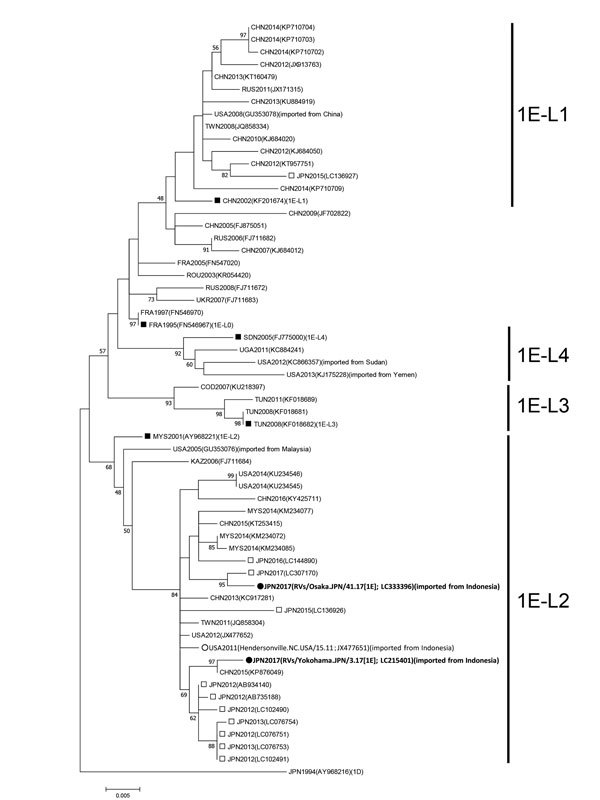
Maximum-likelihood phylogram of the molecular window region (739 nt) within the E1 gene of rubella virus genotype 1E from a 29-year-old man in Japan who had traveled to Indonesia (black circles). We constructed a phylogenetic tree using 61 strains, including the genotype reference strains and the candidate lineage reference strains, using MEGA version 7.0 (http://www.megasoftware.net) and the Tamura-Nei model. Numbers at nodes indicate the bootstrap support values, given as a percentage of 1,000 replicates (values <45 are omitted). The genotype 1D reference strain (RVi/Saitama.JPN/0.94/[1D]) is included as an outgroup. White circles indicate the genotype 1E strain detected in patients returning to the United States from Indonesia in 2011. Black squares indicate candidate genotype 1E lineage reference strains and genotype 1E strains. White squares indicate the strains detected in Japan from 2012–2017. Each strain identification consists of a 3-letter country name abbreviation and detection year. Accession numbers are shown in parentheses. Scale bar indicates nucleotide substitutions per site. COD, Democratic Republic of the Congo; FRA, France; JPN, Japan; KAZ, Kazakhstan; CHN, China; MYS, Malaysia; ROU, Romania; RUS, Russia; SDN, Sudan; TWN, Taiwan; TUN, Tunisia; UGA, Uganda; UKR, Ukraine; USA, United States of America.

To verify rubella elimination, interruptions in transmission of indigenous or imported rubella virus strains must be confirmed through effective surveillance systems (*9*). However, it is difficult to distinguish imported strains from endemic strains and to confirm the control status on the basis of genotype information because the genotypes of global epidemic strains converge to genotypes 1E and 2B (*3*,*7*,*8*,*10*). Therefore, several studies were conducted to subdivide the genotypes on the basis of detailed phylogenetic analysis (*7*,*8*). We reported that a large epidemic in Japan in 2013 might have occurred due to the transport of multiple lineages of rubella virus from rubella-endemic countries (*7*). According to the National Epidemiological Surveillance of Infectious Diseases (NESID) of Japan, during 2015–2017, ≈100 cases of rubella, which is a notifiable disease in Japan, were reported annually (*5*), and genotype 1E strains, including a strain closely related to RVs/Osaka.JPN/41.17[1E], were detected. Although these strains might have been transported from countries with endemic rubella, their origin remains unclear because of insufficient genomic information. 

Japan has a high risk for subsequent rubella epidemics because the proportion of persons susceptible to rubella virus (≈9.0%) has not changed since 2013. In addition, an epidemic can occur when rubella virus is transported from rubella-endemic countries and the infection occurs in susceptible populations, as happened in Japan in 2013. Of the 11 imported cases of rubella to Japan reported in 2017, 4 were from Indonesia, according to the NESID of Japan.. In the case we describe, we identified the rubella-exporting country and clarified the genetic information of the strain, which may contribute to countermeasures for worldwide importation of rubella virus. Rubella control by 2020 is the flagship goal of the World Health Organization South-East Asia region. Indonesia is conducting rubella immunization campaigns targeting ≈70 million children in 2017–2018. Therefore, constructing effective surveillance systems, accumulating genetic information, and promoting immunization in rubella-endemic countries are steps toward the global elimination of rubella.
